# An Unusual Case: *Salmonella* UTI and Orchitis in HIV Patient

**DOI:** 10.1155/2015/216720

**Published:** 2015-07-14

**Authors:** Sabrina Arshed, Hongxiu Luo, John Middleton, Abdalla Yousif

**Affiliations:** Raritan Bay Medical Center, Internal Medicine Residency Program, 530 New Brunswick Avenue, Perth Amboy, NJ 08861, USA

## Abstract

Salmonellosis is a major cause of gastroenteritis in the United States; however, nontyphoidal strains of *Salmonella* have also been known to cause urinary tract infections, usually transmitted via the fecal-urethral route. This can lead to critical illness in those patients with immune deficiencies, especially HIV, cancer patients, and those with diabetes mellitus. However, the spread of the infection from the urinary tract to involve the testicles and epididymis is very rare. Here, we present the first documented case of an immune-compromised young male with a urinary tract infection with orchitoepididymitis.

## 1. Introduction

Salmonellosis is a major cause of morbidity in the United States, with an estimated 800,000–2 million cases annually, typically presenting with gastrointestinal symptomology leading to diarrhea, fever, and abdominal cramping, often quite serious, especially in the elderly, and being immune-compromised, septicemia, or enteric fever. There are a number of less well-known strains of nontyphoidal* Salmonella* that cause extraintestinal disease, such as urinary tract infection (UTI), however, rarely leading to orchitis/epididymitis, as in our case. Nontyphoidal strains of* Salmonella* are more frequently seen in those with immune deficiencies, including HIV and severe malnutrition.

## 2. Case

We present a case of a 46-year-old Caucasian male presented to the emergency department with right scrotal swelling and pain with subjective fever for 2 days. The patient is homosexual and was recently diagnosed with human immunodeficiency virus (HIV) and started on Atripla 2 weeks prior to presentation. It is of note that the patient had a* Salmonella* urinary tract infection (UTI) one week prior to admission, which was confirmed with urine culture and treated with oral cefuroxime 500 mg bid for 5 days.

On physical examination, temperature was 98.7, pulse 87/min, blood pressure 116/77 mmHg, and respiration 20/min. The right side of the scrotum was edematous with erythema and exquisite tenderness. Laboratory findings were as follows: WBC 5.7, hemoglobin 11.2, platelet 272, CD4 246, creatinine 1.4, BUN 29, ALT 14, AST 17, and glucose 97.

The patient underwent emergent scrotal-extrascrotal exploration surgery and was found to have a right scrotal abscess with a hypoplastic testicle; subsequently he underwent abscess drainage and right-sided orchiectomy. The drainage sample culture showed* Salmonella* serotype Typhimurium, susceptible to ceftriaxone, levofloxacin, and Bactrim, but resistant to ampicillin. Abdominal computed tomography (CT) and ultrasound did not show any intra-abdominal abscess, enteroureteral fistula, or pyelonephritis. The patient was discharged with intravenous ceftriaxone for 4 weeks, following up with urologist and infection disease specialist as outpatient.

## 3. Discussion

*Salmonella* infections have a variable presentation; however, the most common is gastroenteritis, which occurs in about 68% of cases, followed by enteric fever (8.8%), often presenting as “FUO,” focal manifestations (7.4%), and asymptomatic carriers (15.8%) [1]. Focal extraintestinal infections are uncommon and usually follow complications with bacteremia.

Orchitis and testicular abscess due to nontyphoid salmonellosis are very rare worldwide. In a review of more than 700 cases of extraintestinal infections caused by* Salmonella*, orchitis and/or epididymitis accounted for only 12 cases (1.4%) [2]. Since then only two cases were reported as epididymoorchitis and testicular abscesses associated with nontyphoid* Salmonella* in adults [3]. The risk factors involved in the development of* Salmonella* orchitis and testicular abscess include being immune-compromised, SLE, and diabetes [3]. Our case is the first documented case of* Salmonella* UTI and orchitis in HIV patient.

There are multiple possible infection routes causing epididymoorchitis or testicular abscess. Although the most common one is the hematogenous route, blood cultures may be negative as the bacteremia may be intermittent [4]. The source of bacteremia is usually traced back to the bladder or kidney, like our case. Ascending infection via the urethra and vas deferens is another route of infection, especially noting that in our case the patient is homosexual. It is fair to reason that he may have acquired this UTI via the rectum-urethra pathway from his partner.

The management of this condition requires intravenous antibiotic therapy and surgical evaluation. Recent guidelines suggest management with surgical exploration, which is performed in the majority of cases because of the strong suspicion of incarcerated hernia or spermatic cord torsion rather than infection. However, in most of cases orchiectomy is not necessary because the conditions are amenable to conservative antibiotic therapy. In our case, the patient did not improve clinically or by ultrasonography after intravenous ceftriaxone, and during scrotal exploration he was found to have large amount of pus in scrotum, as well as a hypoplastic right testicle, which could not be salvaged, thus posing orchiectomy as a necessity in this case.

The major challenge is determining antibiotic course. According to the literature, antibiotic treatment of* Salmonella* UTI is fraught with difficulty due to the high frequency of complicating conditions. In a study of 19 cases, two patients were found to have a recurrence after prolonged treatment (more than two weeks) [5]. These cases show that UTI due to nontyphoid salmonellosis can be recurrent despite prolonged antibiotic treatment. For* Salmonella* orchitis, parenteral therapy is recommended for at least 7 days followed by oral antibiotics for at least 4 weeks [6].

## 4. Conclusion

Nontyphoidal strains of* Salmonella* present potentially increased morbidity for patients with immune deficiencies, including HIV, diabetes, severe malnutrition, and SLE. Although* Salmonella* UTI is uncommon, a concomitant orchitis/epididymitis proves to be very rare, which has the potential to botch patient care. Furthermore, the management is difficult, given the high incidence of recurrence despite long-term parenteral antibiotic therapy. It is imperative for clinicians to keep in mind the variable presentation of salmonellosis, especially in the immune-compromised patient population, where nontyphoidal strains of* Salmonella* may lead to serious illness.
